# Mental health evaluation of patients with Inflammatory Bowel Disease and psychiatric comorbidities during the COVID-19 pandemic

**DOI:** 10.1192/j.eurpsy.2022.739

**Published:** 2022-09-01

**Authors:** S. Vanzetto, M. Vismara, A. Frediani, N. Cassina, G. Maconi, M. Bosi, C. Viganò, B. Dell’Osso

**Affiliations:** 1 Luigi Sacco University Hospital, Psychiatry 2 Unit, ASST FBF-Sacco, University Of Milan, Milan, Italy; 2 “Aldo Ravelli” Center for Nanotechnology and Neurostimulation”, University Of Milan, Milan, Italy; 3 Gastroenterology Unit, Department of Biomedical and Clinical Sciences, “l. Sacco” Hospital, Milan, Italy; 4 Department of Psychiatry and Behavioral Sciences, Stanford University, Milan, Italy; 5 “Centro per lo studio dei meccanismi molecolari alla base delle patologie neuro-psico-geriatriche”, University Of Milan, Milan, Italy

**Keywords:** Psychiatric comorbidities, Inflammatory Bowel Disease, Mental health evaluation, Covid-19 pandemic

## Abstract

**Introduction:**

The mental health of subjects with chronic medical illnesses, such as Inflammatory Bowel Disease (IBD- Crohn’s Disease and Ulcerative Colitis), is typically compromised and the current COVID-19 pandemic might have additionally increased this burden.

**Objectives:**

The aim of the present study was to investigate, during the COVID-19 pandemic, if the presence of a comorbid psychiatric disorder has played a role as an aggravating factor on mental health in patients with IBD.

**Methods:**

Twenty Five patients with psychiatric comorbidities (PC+) and twenty five without (PC-) comparable for age and gender, were recruited at the Gastroenterology department at Sacco University Hospital in Milan. Participants were assessed a psychiatric evaluation, collecting socio-demographic variables and measures of anxiety and depression [on the Hospital Anxiety Depression Scale (HADS)], sleep patterns [on the Insomnia Severity Index (ISI)] and general health status [on the Short Form Health Survey 36 (SF-36)].

Comparative statistical analyses were performed with t test with Bonferroni correction.

**Results:**

PC+ (n=25) showed more severe anxiety and depressive symptoms compared with PC- (n=25) (p <.001) and worse sleep pattern (p<.05). With respect to general health status, PC+ showed reduced physical activities (p<.05), social activities (p<.05), mental health (p<.01) and role limitations due to physical health (p<.05).

**Conclusions:**

The present findings showed a worse mental health in subjects with IBD and psychiatric comorbidities during Covid-19 pandemic, highlighting the importance of screening and treatment of psychiatric symptoms disorders in these patients.

**Disclosure:**

No significant relationships.

